# Biomarkers for detecting malignant pleural mesothelioma: Protocol for a reanalysis of published data based on systematic reviews of diagnostic test accuracy

**DOI:** 10.1097/MD.0000000000016028

**Published:** 2019-06-14

**Authors:** Xiangyi Zan, Yuping Wang, Junnian Shi, Lanting Zhao, Yan Zhao, Rong Liu, Yongning Zhou, Yixin Wan

**Affiliations:** a Department of Respiratory, Lanzhou University Second Hospital, Lanzhou University Second Clinical Medical College; b Key Laboratory for Gastrointestinal Diseases of Gansu Province, Lanzhou University; c Department of Gastroenterology, First Hospital of Lanzhou University, Lanzhou University First Clinical Medical College, Lanzhou, China.

**Keywords:** adjusted indirect comparison, biomarker, diagnostic test accuracy, malignant pleural mesothelioma, systematic reviews

## Abstract

**Background:**

Malignant pleural mesothelioma (MPM) is a highly invasive tumor caused primarily by asbestos exposure. In recent decades, the incidence of MPM has shown an increasing trend, posing a great threat to human health. Although there is currently no effective way to treat MPM, patients can survive for more than 5 years if the tumor is removed early. Several systematic reviews (SRs) have evaluated the diagnostic value of biomarkers for diagnosing MPM. However, no studies have been conducted to analyze the quality of these SRs and it remains unclear which biomarker is the excellent diagnostic test. This study aims to assess the methodological quality of the SRs and reanalyze the published data based on SRs to find the optimal biomarker for the early diagnosis of MPM.

**Methods:**

A systematic search will be performed in PubMed, Embase.com, the Cochrane Library of Systematic Reviews, and Web of Science to identify SRs reporting value of biomarkers for detecting MPM. We will evaluate the risk of bias of the included SRs according to the Assessment of Multiple Systematic Reviews-2 (AMSTAR-2) instrument. Standard pairwise meta-analysis and adjusted indirect comparison will be used to compare the diagnostic value of different biomarkers.

**Results:**

The results of this study will be submitted to a peer-reviewed journal for publication.

**Conclusion:**

This study will reanalyze the published data based on SRs to find a biomarker with the superior diagnostic performance for the diagnosis of MPM.

**Ethics and dissemination:**

Ethics approval and patient consent are not required as this study is an overview based on published systematic reviews.

**PROSPERO registration number:**

CRD42019125880.

## Abstract 2

Supplemental Digital Content is available in the text

## 1 Introduction

Malignant mesothelioma, a highly invasive tumor caused primarily by asbestos exposure, is primarily derived from the surface serosal cells of the pleura and, to a lesser extent, from the peritoneum, pericardium, and vaginal lining.^[1–4]^ Mesothelioma is usually divided into 3 major histological subtypes: epithelial, sarcoma, and biphasic, with incidence rates of 60%, 10%, and 30%, respectively.^[1,5,6]^ In recent decades, the occurrence of malignant pleural mesothelioma (MPM) has shown an increasing trend. It is estimated that between 2010 and 2020, about 1000 people will die of MPM every year, and the highest peak will occur in 2012 to 2025, with a maximum of 800 deaths per year in males.^[7–10]^ Currently, there is no effective treatment for MPM, with a median overall survival of 12 to 18 months.^[3,11]^ However, patients with early disease can survive for more than 5 years if the tumor is rapidly removed.^[12]^ Therefore, sensitive biomarkers are increasingly needed to help early diagnosis and management of MPM.

The primary diagnostic method of MPM is the histopathological evaluation of the pleural biopsy. However, this method requires thoracoscopic surgery or thoracotomy, which is very harmful to the patient and its sensitivity is not high.^[3–6]^ Therefore, tumor biomarkers are becoming more attractive due to their non-invasive characteristics and relatively inexpensive.^[2]^ Over these years, several diagnostic biomarkers, including fibulin-3, DNA, microRNAs, and antibodies have been explored for early detection of MPM.^[3,13,14]^ And some systematic reviews (SRs) have evaluated the diagnostic value of these biomarkers.^[15–17]^ If these SRs were carried out well, they could provide the best evidence for clinical practice.^[18–21]^ However, no studies have been conducted to analyze the quality of these SRs. Furthermore, it remains unclear which biomarker is the excellent diagnostic test for early and accurate detection of MPM. Thus, this study aims to assess the methodological quality of the SRs and reanalyze the published data based on SRs for the biomarkers to find the optimal biomarker for the early diagnosis of MPM.

## 2 Methods

We will reanalyze the published data of systematic reviews of diagnostic test accuracy for MPM. This research protocol will fully follow the Preferred Reporting Items for Systematic Reviews and Meta-analysis Protocols (PRISMA-P) checklist.^[22]^ This project has been registered on international prospective register of systematic review (PROSPERO) (CRD42019125880).

### 2.1 Criteria for considering studies for this review

#### 2.1.1 Type of studies

To be included in this overview, SRs must include meta-analytical results and meet the participants, index tests, and outcomes of interest criteria described below. We will exclude SRs that only report data narratively.

#### 2.1.2 Participants

Patients diagnosed with MPM according to pathological histology examination. We will not include people with distant metastasis of MPM. We will not put restrictions on age, race, sex, and nationality of participates, as well as treatment plan and stage of cancer.

#### 2.1.3 Index tests

Single biomarker or combined biomarkers used for the diagnosis of MPM is considered eligible for this overview. There are no restrictions on the types of biomarkers but 1 biomarker combines with imaging modalities or other index tests will be excluded.

#### 2.1.4 Outcome measures

SRs should report the diagnostic value of sensitivity, specificity or diagnostic odds ratio (DOR) and their 95% confidence interval (CI) for each included primary study. If the sensitivity, specificity, and DOR were not reported, the SRs provided the true positive, false positive, true negative, and false negative values which allow us to calculate the diagnostic performance indices for each primary study will also be included.

#### 2.1.5 Exclusion criteria

(1) SRs that did not report the diagnostic value of biomarkers.
(2) SRs without meta-analysis.
(3) Publications with incomplete data.
(4) Conference abstracts, review articles, guidelines, consensus, documents or expert position papers, comments, letters, brief reports, proceedings, or protocol studies.

### 2.2 Search methods for identification of studies

The search strategies for relevant SRs were discussed by the review team and were established in co-operation with an experienced medical information specialist.^[23]^ We conducted electronic searches in PubMed, the Cochrane Library, Embase.com, and Web of Science to identify relevant SRs from inception to February 2019. We applied no language or publication status restrictions. The search strategy of the PubMed is presented in Supplementary 1.

### 2.3 Selection of studies

Two authors will independently screen the titles and the abstracts of every study after the search is completed. We will acquire all the articles deemed to be suitable by either author in full text for further assessment. Then, the same 2 authors will evaluate potential full texts and select the studies in accordance with the inclusion/exclusion criteria. The authors will then confer and agree upon the studies for inclusion, resolving any disagreements by discussion and consensus. If an agreement cannot be reached, a third reviewer will be consulted.

### 2.4 Data extraction and management

Two reviewers will independently extract data from the included SRs according to a predesign Microsoft Excel sheet. We will retrieve the following data:

(1) General information: author, country of the corresponding author, number of authors, publication year, journal name, country of the journal, funding, and types of included studies.
(2) Sample size: number of included studies, and number of participants.
(3) Baseline characteristics: baseline diagnosis, age, sex, and location (country, state, region).
(4) The index tests: number and name of biomarkers.
(5) Reference standard.
(6) Data of sensitivity, specificity, likelihood ratio, DOR, the area under the curve, and their 95% CI of each original study included in the SRs.

If we identify multiple reviews addressing the same research question but share the same primary study, the data of the overlapping original studies will only be included once. For updated original studies, the most updated study will be selected for data extraction, while the older versions will be used as supplementary information, if necessary. If the SRs did not provide the diagnostic performance indices for each original study, the number of true positive, false positive, true negative, false negative will be used to calculate the sensitivity, specificity, and DOR. We will contact study authors for missing or unclear data. Discrepancies will be resolved by consensus between 2 reviewers. A third reviewer will be invited for consensus adjudication if discrepancy is not resolved.

### 2.5 Assessment of methodological quality

The methodological quality of included SRs will be assessed according to the Assessment of Multiple Systematic Reviews-2 (AMSTAR-2) instrument. This updated version of the original AMSTAR tool allows for the appraisal of systematic reviews of randomized and non-randomized studies of interventions.^[24–27]^ It contains 16 items, among which 7 are critical domains. The overall confidence of the results of the review will be classified as high, moderate, low, and critically low, and each item will be responded to “Yes”, “No”, or “Partial Yes”. The quality of SRs will be assessed by 1 reviewer and verified by another. Disagreements will be resolved by consensus or third-party adjudication if consensus cannot be reached.

### 2.6 Statistical analysis and data synthesis

#### 2.6.1 Pairwise meta-analysis

We will use the data of sensitivity, specificity, DOR, positive likelihood ratio, negative likelihood ratio, and their 95% CI lower limit, 95% CI upper limit extracted from each original study of the SRs to perform the pairwise meta-analysis. The forest plots will be generated to present the diagnostic indices for each biomarker. The heterogeneity between studies will be evaluated with the Chi-squared test and determined using the I^2^ value. If the I^2^ is less than 50%, the statistical heterogeneity between tests can be ignored, and the effect size will be estimated using a fixed-effect model. If we find considerable heterogeneity among the studies, we will conduct subgroup analyses to explore the sources of heterogeneity. If there is no clinical heterogeneity, the random effects model will be used to perform the meta-analysis. Otherwise, clinical heterogeneity will be explored through discussion with the review team. We will conduct the analyses and generate the plots using STATA (13.0; Stata Corporation, College Station, TX).

#### 2.6.2 Adjusted indirect comparisons

We will first calculate relative sensitivity, relative specificity, and relative DOR between different biomarkers using STATA. Then, the indirect comparisons will be conducted using the relative diagnostic indices.

#### 2.6.3 Subgroup analysis

We will conduct between study subgroup analysis and within study subgroup analysis. We will identify all the primary studies reporting the results of subgroup analysis and extract data from these studies. If sufficient data extracted from the primary studies, we will perform subgroup analyses to explore whether the sex, age, and weight of patients, the country of the study, the treatment plan, and the cutoff of biomarkers will affect the diagnostic value of biomarkers.

### 2.7 Assessment of publication bias

The funnel plot and Egger test will be conducted to detect publication bias if more than 10 SRs reported the diagnostic value of a biomarker.

## Author contributions

XYZ, YNZ, and YXW planed and designed the research. YPW, JNS, LTZ, YZ, and RL tested the feasibility of the study. YNZ and YXW provided methodological advice, polished and revised the manuscript. XYZ, YNZ, and YXW wrote the manuscript. All authors approved the final version of the manuscript.

**Conceptualization:** Xiangyi Zan, Junnian Shi, Yongning Zhou, Yixin Wan.

**Data curation:** Xiangyi Zan, Yuping Wang, Junnian Shi, Yixin Wan.

**Formal analysis:** Xiangyi Zan, Yongning Zhou.

**Funding acquisition:** Yongning Zhou.

**Investigation:** Xiangyi Zan, Yuping Wang, Junnian Shi, Yan Zhao, Rong Liu.

**Methodology:** Yan Zhao, Yongning Zhou, Yixin Wan.

**Project administration:** Yongning Zhou.

**Resources:** Yuping Wang, Junnian Shi, Yan Zhao, Rong Liu.

**Software:** Xiangyi Zan, Yuping Wang, Lanting Zhao.

**Supervision:** Yongning Zhou, Yixin Wan.

**Validation:** Lanting Zhao, Yixin Wan.

**Visualization:** Yuping Wang, Lanting Zhao.

**Writing – original draft:** Xiangyi Zan, Yongning Zhou, Yixin Wan.

**Writing – review & editing:** Xiangyi Zan, Yongning Zhou, Yixin Wan.

## Supplementary Material

Supplemental Digital Content
