# Supplementary material for: Biomarkers for detecting malignant pleural mesothelioma: Protocol for a reanalysis of published data based on systematic reviews of diagnostic test accuracy
Source: Medicine (Baltimore). 2019 Jun 14;98(24):e16028. doi: 10.1097/MD.0000000000016028 (PMC6587538; doi:10.1097/MD.0000000000016028)
Supplement: Supplemental Digital Content [file medi-98-e16028-s001.docx]

**The search strategy of PubMed**

#1 "Mesothelioma"[Mesh] OR "Mesothelioma, Malignant"[Supplementary Concept]

#2 Mesothelioma*[Title/Abstract] OR "submesothalial cancer"[Title/Abstract] OR celothelioma[Title/Abstract] OR mesotheliomatosis[Title/Abstract]

#3 #1 OR #2

#4 "Biomarkers, tumor"[Mesh]

#5 cancer biomarker*[Title/Abstract] OR cancer bio-marker[Title/Abstract] OR cancer marker*[Title/Abstract] OR tumor biomarker*[Title/Abstract] OR tumor bio-marker[Title/Abstract] OR tumor marker*[Title/Abstract] OR tumour biomarker*[Title/Abstract] OR tumour bio-marker[Title/Abstract] OR tumour marker*[Title/Abstract] OR carcinogen biomarker*[Title/Abstract] OR carcinogen bio-marker[Title/Abstract] OR carcinogen marker*[Title/Abstract] OR neoplasm biomarker*[Title/Abstract] OR neoplasm bio-marker[Title/Abstract] OR neoplasm marker*[Title/Abstract] OR neoplasm metabolite marker*[Title/Abstract] OR tumor metabolite marker*[Title/Abstract] OR cancer metabolite marker*[Title/Abstract] OR tumour metabolite marker*[Title/Abstract] OR carcinogen metabolite marker*[Title/Abstract]

#6 #4 OR #5

#7 "Sensitivity AND Specificity"[Mesh] OR "False Positive Reactions"[Mesh] OR "False Negative Reactions"[Mesh] OR "ROC Curve"[Mesh] OR "Predictive Value of Tests"[Mesh]

#8 sensitivity[Title/Abstract] OR specificity[Title/Abstract] OR "receiver operating characteristic"[Title/Abstract] OR "receiver operator characteristic"[Title/Abstract] OR "predictive value*"[Title/Abstract] OR roc[Title/Abstract] OR "pre-test odds"[Title/Abstract] OR "pretest odds"[Title/Abstract] OR "pre-test probability*"[Title/Abstract] OR "pretest probability*"[Title/Abstract] OR "post-test odds"[Title/Abstract] OR "posttest odds"[Title/Abstract] OR "post-test probabilit*"[Title/Abstract] OR "posttest probabilit*"[Title/Abstract] OR "likelihood ratio*"[Title/Abstract] OR "positive predictive value*"[Title/Abstract] OR "negative predictive value*"[Title/Abstract] OR "false negative*"[Title/Abstract] OR "false positive*"[Title/Abstract] OR "true negative*"[Title/Abstract] OR "true positive*"[Title/Abstract]

#9 #7 OR #8

#10 "Meta-Analysis as Topic"[Mesh] OR "Meta-Analysis"[Publication Type]

#11 meta analysis[Title/Abstract] OR meta analyses[Title/Abstract] OR meta-analysis[Title/Abstract] OR meta-analyses[Title/Abstract] OR metaanalysis[Title/Abstract] OR metanalysis[Title/Abstract] OR met-analysis[Title/Abstract] OR metaanalyses[Title/Abstract] OR metanalyses[Title/Abstract] OR met-analyses[Title/Abstract] OR data pooling[Title/Abstract] OR data poolings[Title/Abstract] OR clinical trial overview[Title/Abstract] OR clinical trial overviews[Title/Abstract] OR systematic review[Title/Abstract] OR systematic reviews[Title/Abstract]

#12 #10 OR #11

#13 #3 AND #6 AND #9 AND #12
